# Electrically controllable sudden reversals in spin and valley polarization in silicene

**DOI:** 10.1038/srep33701

**Published:** 2016-09-20

**Authors:** Qingtian Zhang, K. S. Chan, Jingbo Li

**Affiliations:** 1 School of Materials and Energy, Guangdong University of Technology, Guangzhou, Guangdong 510006, People’s Republic of China; 2 Department of Physics and Materials Science, City University of Hong Kong, Tat Chee Avenue, Kowloon, Hong Kong, People’s Republic of China; 3 City University of Hong Kong Shenzhen Research Institute, Shenzhen, 5183000, People’s Republic of China

## Abstract

We study the spin and valley dependent transport in a silicene superlattice under the influence of a magnetic exchange field, a perpendicular electric field and a voltage potential. It is found that a gate-voltage-controllable fully spin and valley polarized current can be obtained in the proposed device, and the spin and valley polarizations are sensitive oscillatory functions of the voltage potential. In properly designed superlattice structure, the spin and valley polarizations can be reversed from −100% to 100% by a slight change in the external voltage potential. The energy dispersion relations of the superlattice structure are also investigated, which helps us to understand the effects of the superlattice structure. The switching of the spin direction and the valley of the tunneling electrons by a gate voltage enables new possibilities for spin or valley control in silicene-based spintronics and valleytronics.

Silicene, a close relative of graphene, is a monolayer honeycomb lattice structure of silicon, which has been intensively studied since it was successfully synthesized in laboratories^1^,^2^,^3^. Unlike the strictly two-dimensional graphene, silicene has a buckled structure, and the two sublattice atomic planes are formed by the A and B atoms, with a vertical separation distance of 0.46 Å^4^,^5^. Owing to this vertical separation between the A and B sublattices, a perpendicular electric field *E*_*z*_ applied to the silicene induces different onsite energies in the A and B sublattices and a staggered sublattice potential (*D*_*z*_ = *E*_*z*_*d*)^6^,^7^,^8^. The staggered sublattice potential plays important roles in valley-dependent transport in honeycomb strutures, and induces quantum Hall phase transitions in silicene according to the study by Ezawa^9^,^10^; it is as well an effective method to control the transport properties of silicene^11^,^12^. According to theoretical prediction, silicene has a large intrinsic spin orbit interaction (SOI) compared to graphene, and the value of intrinsic SOI is Δ_SO_ ≈ 1.55–7.9 meV^5^,^13^. So, there is an energy gap between the conduction band and valence band in silicene, which makes it a quantum spin Hall insulator^14^. Furthermore, silicene should be relatively easy to be integrated with the current silicon-based nanotechnology.

As in graphene, there is also a valley degree of freedom for electrons in silicene arised from the honeycomb lattice structure. The concept of valley is used in graphene to describe the two inequivalent Dirac cones at the K and K’ points in the hexagonal Brillouin zone^15^,^16^. As we all know, the two intrinsic characteristics of an electron, charge and spin, are used to carry and process information in electronics and spintronics^17^,^18^,^19^,^20^,^21^,^22^. Similarly, the valley degree of freedom can also be used to carry and process information, and this area of study is referred to as valleytronics^23^,^24^,^25^,^26^ which has attracted a lot of research interest recently^27^,^28^,^29^,^30^.

It is well known that the superlattice structures are very successful in controlling the transport properties of semiconductors^31^,^32^,^33^,^34^ and graphene^35^,^36^,^37^,^38^,^39^,^40^,^41^. Presently the graphene superlattices have been realized in the experiments^42^,^43^, while relatively little work has been carried out on the spin and valley transport in silicene superlattice^44^,^45^. In the present work, we study ballistic spin and valley transport and the band structure of Dirac electrons in silicene under the modulation of a superlattice of ferromagnetic stripes. Our study is different from the previous study^45^ which is mainly about the diffusive transport properties. It is found that the transmission probabilities for all spin and valley flavors exhibit different transmission features, which result in full spin and valley polarization in our proposed device. Moreover, we predict an electrically reversible spin and valley polarization in the proposed silicene superlattice. We note that the spin and valley polarizations are sensitive oscillatory functions of external gate voltages, and the magnitude of spin and valley polarizations can be suddenly switched from +100% to −100% by slight changes in the external gate voltages. The spin and valley polarization switching effects could have useful applications in the development of silicene spintronics and valleytronics, such as spintronics (valleytronics) multiple-value logic devices.

The system under consideration is sketched in Fig. 1. In Fig. 1(a), we show a superlattice of ferromagnetic stripes on top of the silicene layer. The ferromagnetic stripes can induce an exchange spin splitting in silicene through the proximity effect. In the previous study, Yokoyama^46^ has investigated the valley and spin dependent transport in silicene with a single magnetic barrier, in the presence of the exchange splitting induced by a ferromagnetic stripe and the staggered potential created by a perpendicular electric field. In this study, the exchange spin splitting and staggered potential are both considered, and the ferromagnetic stripes are also used as top gates for adding electric potentials. In Fig. 1(b), we show the side view of the silicene sublattices, A and B, which are separated by a perpendicular distance 2d. So, a perpendicular electric field applied to the silicene plane induces a staggered sublattice potential term. In the system studied, the superlattice structure is connected to two silicene leads without exchange splitting, staggered potential and gate voltage.

In some previous studies, the exchange splitting in graphene induced by the ferromagnetic stripe have been calculated theoretically. Haugen *et al*. considered the proximity effects of ferromagnetic insulator EuO, and the exchange splitting is estimated to be 5 meV^47^. Yang *et al*. calculated the exchange splitting induced by proximity of ferromagnetic insulator EuO through First-Principles, and their numerical results show that the exchange splitting can be up to 36 meV^48^. The exchange splitting is chosen to be 36 meV in our numerical calculations, but we also examine the effects of the exchange splitting in the value region: 0~50 meV.

## Methodology

The low energy electrons in silicene are described by the Dirac-like Hamiltoonian, in the presence of exchange splitting, gate voltage and perpendicular electric field, as^49^


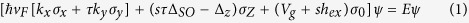


where *v*_*F*_ ≈ 5.5 × 10^5^*ms*^−1^ is the Fermi velocity, 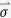 are the Pauli matrices for pseudospin in the sublattice space, *σ*_0_ is the identity matrix, 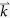 is the wave vector, *s* = +1 and −1 labels the spin up and down respectively, *τ* = +1 and −1 labels the K and K’ valleys respectively, and Δ_*SO*_ is the intrinsic SOI. And the other terms are externally added parameters: Δ_*Z*_ is the staggered sublattice potential induced by the external electric field, *V*_*g*_ is the gate voltage applied through the ferromagnetic stripe, and *h*_*ex*_ is the exchange splitting induced by the ferromagnetic stripe.

The wave functions in each region for an electron incident from the left into a single barrier of the superlattice structure can be written as


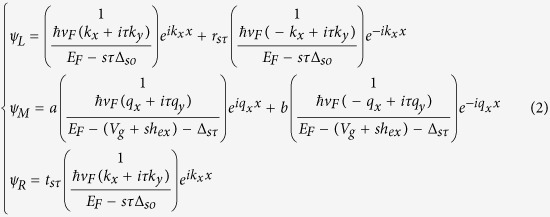


where *ψ*_*L*_ and *ψ*_*R*_ are the wave functions in the left and right regions respectively, and *ψ*_*M*_is the wave function in the barrier region. And the parameter Δ_*sτ*_ is defined as Δ_*sτ*_ = *sτ*Δ_*so*_ − Δ_*z*_. The wave vectors satisfy the energy dispersion relations 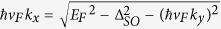 and 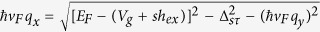. Here *q*_*y*_ = *k*_*y*_ due to the translational invariance in the y direction. The transmission amplitude *t*_*τs*_ and reflection amplitude *r*_*τs*_ are obtained by matching the wave functions across the left and right boundaries. For the whole superlattice structure, the total transmission and reflection amplitudes are obtained by combining the scattering matrices using the expression given in pages 125–6 of the ref. 50. In our numerical calculations, the number of superlattice barriers is 20. The spin and valley resolved conductance is written as


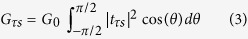


where 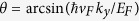 is the incident angle measured from the x direction, and 
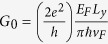
 with *L*_*y*_ being the sample size along the y direction. The conductance obtained in our numerical calculation is expressed in the unit of *G*_*0*_ as shown in Eq. (3) throughout the paper. The valley resolved conductance is defined as *G*_*K*(*K*′)_ = *G*_*K*(*K*′)↑_ + *G*_*K*(*K*′)↓_, and the spin resolved conductance is defined as *G*_↑(↓)_ = *G*_*K*↑(↓)_ + *G*_*K*′↑(↓)_. So, we can define the spin polarization *P*_*S*_ and valley polarization *P*_*V*_ as


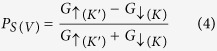


For our proposed superlattice structure, the electronic dispersion, based on the Bloch’s theorem, is determined by^36^,^51^


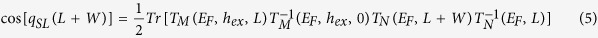


where *q*_*SL*_ is the Bloch wave vector along the periodic direction for the silicene superlattice. The two matrices *T*_*M*_and *T*_*N*_ are the transfer matrices for the barrier region and normal silicene region respectively as given below


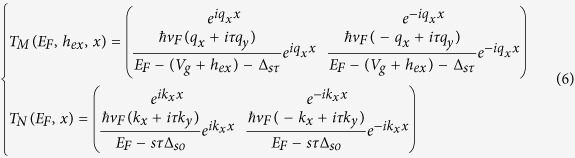


Usually, we can use Eq. (5) and Eq. (6) to plot the relation between the energy *E*_*F*_ and the wave vector *q*_*SL*_ and *k*_*y*_, which is very useful for understanding the transport properties of the superlattice structure^36^,^40^,^51^. However, in this study, we are interested in how the gate voltage *V*_*g*_ can be used to control the ballistic properties, and we therefore plot values of *V*_*g*_ for which Eq. (5) is satisfied with a fixed value of *q*_*SL*_ and a fixed value of *E*_*F*_, which means there is a propagating electron state in the superlattice with real wave vector *q*_*SL*_ at energy *E*_*F*_. In these plots we can find ranges of *V*_*g*_ in which electrons can propagate in the superlattice structure. These plots are useful to understand the relation between the gate voltage *V*_*g*_ and the transmission properties and help to design a structure with specific characteristics.

## Results and Discussion

**Figure 2:**
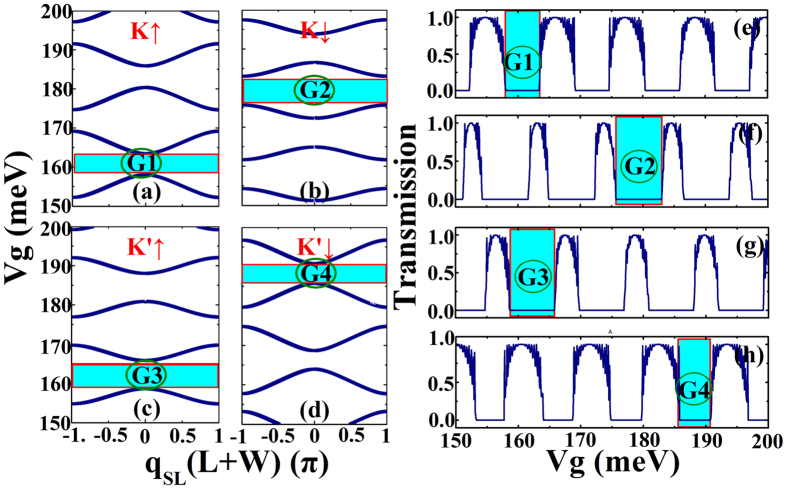
(**a–d**) Are *V*_*g*_ vs *q*_*SL*_ for a fixed value of *k*_*y*_ and *E*_*F*_ of the electrons for all spin and valley flavors in the proposed superlattice structure. (**e–h**) Are transmission for all spin and valley flavors from top to bottom:*T*_*K*↑_ *T*_*K*↓_ *T*_*K*′↑_ *T*_*K*′↓_. The parameters are: Δ_*SO*_ = 3.9 *meV*, *E*_*F*_ = 5 *meV*, *h*_*ex*_ = 36 *meV*, Δ_*z*_ = 40 *meV*, *L* = 100 nm, *W* = 200 nm and *k*_*y*_ = 0.

From Eq. (5) it is clear that for a given *k*_*y*_ and *E*_*F*_ we can plot the relation between the external gate voltage and Bloch wave vector *q*_*SL*_. These relations between *V*_*g*_ and *q*_*SL*_ are shown in Fig. 2(a–d), which is obtained numerically from Eq. (5). It is found that gaps appear in this *V*_*g*_ − *q*_*SL*_ relation, where there is no real value of q for the *V*_*g*_ considered, when it is modulated by changing the gate potential *V*_*g*_. For example, there are four gaps in Fig. 2(a), in which the electrons are blocked, and the corresponding transmission probabilities are zero in these ranges. To be specific, we choose one gap from each figure in Fig. 2(a–d)), and they are all marked by a blue square. The corresponding transmission gaps are also marked in Fig. 2(e–h), and it is obvious that transmissions in the gaps are all zero. Moreover, electrons for different valley and spin flavors have different *V*_*g*_ − *q*_*SL*_ relations, so we predict that interesting features of spin and valley polarizations can be found in properly designed superlattice structure.

**Figure 3:**
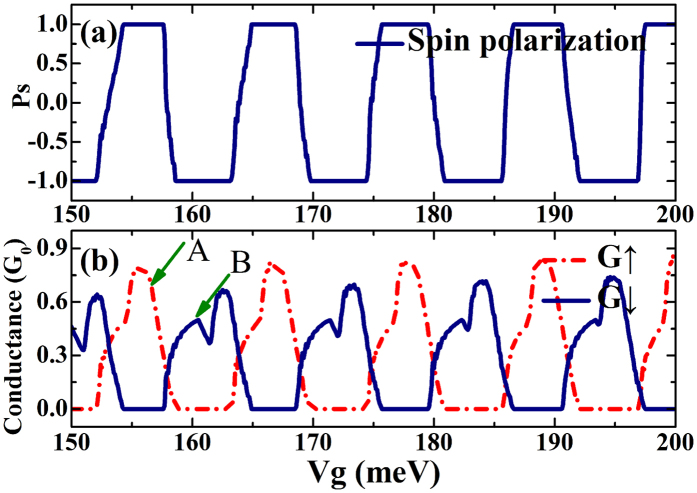
(**a**) Spin polarization for 20 superlattice barriers plotted as a function of externally applied gate voltage. (**b**) Spin resolved conductance *G*_↑_ and *G*_↓_ plotted as a function of externally applied gate voltage. The parameters are: Δ_*SO*_ = 3.9 *meV*, *E*_*F*_ = 5 *meV*, *h*_*ex*_ = 36 *meV*, Δ_*z*_ = 40 *meV*, *L* = 100 nm and *W* = 200 nm.

We present the spin dependent transport properties for our proposed superlattice in Fig. 3. In Fig. 3(a), we show the spin polarization of our proposed structure as a function of the external gate voltages and note that the spin polarization can be reversed by a slight change in the gate voltage. Moreover, the spin polarization is changed rapidly from 100% to −100%, which is thus an effective and sensitive knob for controlling spin polarization direction electrically. For realistic application, it is very important that the proposed structure has measurable conductance. In this regard, we examine the spin resolved conductance *G*_↑_ and *G*_↓_ as a function of the external gate voltage as plotted in Fig. 3(b). Both *G*_↑_ and *G*_↓_ oscillate with the gate voltage, and it is noted in Fig. 3(b) that the adjacent conductance peaks are from different electron spins. For example, the peak marked by arrow A is from the up spin electrons, while in this range of gate voltages the conductance from down spin electrons is zero. Similarly, the peak marked by arrow B is from down spin electrons, and in this range, the conductance from the up spin electrons is found to be zero. We also note that, the conductance shown in Fig. 3 can be measurable in experiment, so our proposed scheme for spin polarization switching is realizable experimentally.

**Figure 4:**
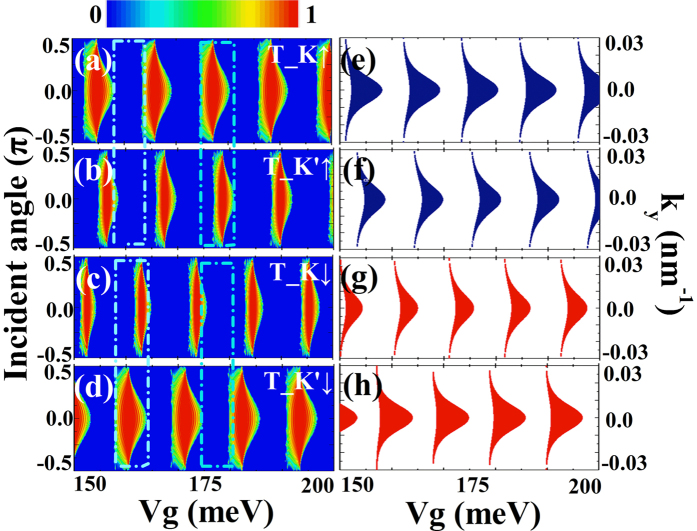
(**a**–**d**) Contour plots of the transmission probability as a function of incident angle and external gate voltage. (**e**,**f**) Dispersion relations of the electrons in silicene modulated by the ferromagnetic superlattice. The colour regions are values of *V*_*g*_ and *k*_*y*_ which have propagating electron states in the superlattice (states with real *q*_*SL*_). Blue is for up spin electrons and red is for down spin electrons. The parameters are the same as that of Fig. 3.

The rapid reversals of spin polarization shown in Fig. 3 can be understood by the transmission probabilities and dispersion relation of the superlattice structure. In the left column of Fig. 4, we show the contour plots of the transmission probabilities as a function of the incident angle and gate voltage for all valley and spin flavors. It is noted that the contour plots of transmissions are in agreement with the conductance shown in Fig. 3. To show the regions, we put dashed squares in the contour plots. The transmission probability for up spin electrons is defined as *T*_↑_ = *T*_*K*↑_ + *T*_*K*′↑_, and it is can be seen from Fig. 4(a,b) that the transmission is almost zero in the marked square around *V*_*g*_ = 160 meV. However, the transmission probability for down spin electrons *T*_↓_ = *T*_*K*↓_ + *T*_*K*′↓_ is high in the marked square. So, we have spin polarization *P*_*s*_ = −100%in this marked square, which is related to the peak shown in Fig. 3(b) marked by arrow B. Comparing the left column with the right column of Fig. 4, we find that the contour of the transmission probability can be correlated with the superlattice bandstructure. So, the spin-dependent transport properties obtained are the features of the proposed superlattice structure.

**Figure 5:**
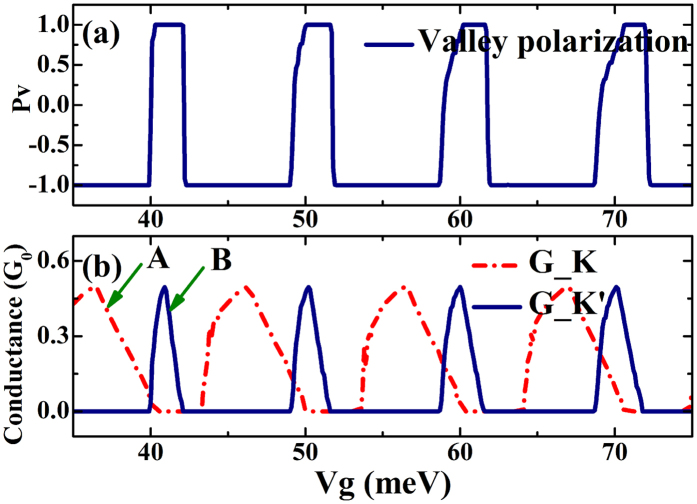
(**a**) Valley polarization for 20 superlattice barriers plotted as a function of the externally applied gate voltage. (**b**) Valley resolved conductance *G*_*K* and *G*_*K*′ plotted as a function of the externally applied gate voltage. The parameters used are: Δ_*SO*_ = 3.9 *meV*, *E*_*F*_ = 5 *meV*, *h*_*ex*_ = 36 *meV*, Δ_*z*_ = 40 *meV*, *L* = 100 nm and *W* = 200 nm.

Figure 5(a) displays the valley polarization*P*_v_, and Fig. 5(b) displays the valley resolved conductance *G*_*K* and *G*_*K*′. As shown in Fig. 5, the superlattice structure proposed in this study can be used to obtain a high valley polarization. It is noted from Fig. 5(a) that the valley polarization is a sensitive oscillatory function of the gate voltage, and the direction of valley polarization can be tuned by the external gate voltages. Rapid reversals of valley polarization can be realized by slight changes in the external gate voltage. We found that the magnitude of valley polarization is switched from +100% to −100% suddenly.

The characteristics shown in Fig. 5(a) can be understood in terms of the valley resolved conductance in Fig. 5(b), in which it is readily noted that the adjacent conductance peaks are from different valleys. For example, *G*_*K*is around 0.5 *G*_0_ in the energy region: 37~40 meV (see the peak marked by arrow A), while *G*_*K*′ is completely 0. However, *G*_*K*′ is not zero in the energy region: 40~42 meV (see the peak marked by arrow B) while G_K is zero. As a result, the valley polarization is reversed from −100% to 100% when we change the gate voltage from one peak to another peak.

**Figure 6:**
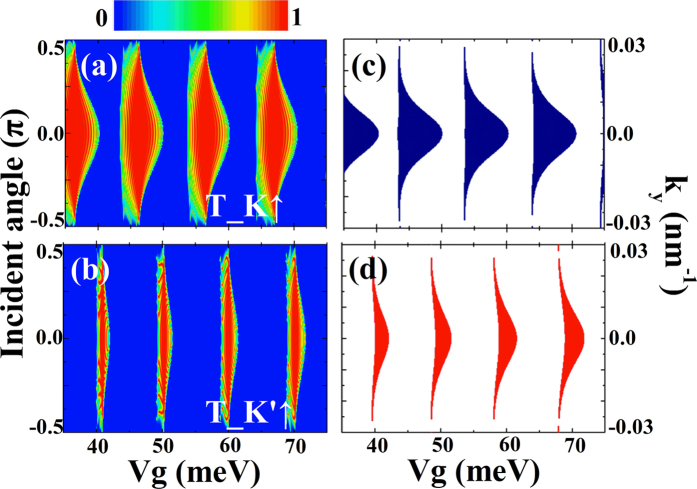
(**a**,**b**) Contour plots of the transmission probability as a function of incident angle and external gate voltage. (**c**,**d**) Dispersion relations of the electrons in silicene modulated by the ferromagnetic superlattice. The parameters are the same as that of Fig. 5.

The features shown in Fig. 5 can be further understood through the transmission probabilities and dispersion relation for all spin and valley flavors. In Fig. 6(a,b) we show the contour plots of the transmission probability as a function of the incident angle and gate voltage for all valley and spin flavors. However, it is noted that only *T*_*K*↑ and *T*_*K*′↑ are shown in Fig. 6, because the transmission probabilities *T*_*K*↓ and *T*_*K*′↓ are zero. We can see from Fig. 6(b) that the reflection is almost perfect for the *K*′↑ electrons in the whole incident angle region for the gate voltage *V*_*g*_ = 45 *meV*, however, we have large transmission probability *T*_*K*↑ in the same gate voltage region (see Fig. 6(a)). So, we have large conductance *G*_*K* in this region but the conductance *G*_*K*′ is zero. In Fig. 6(a,b), it is noted that high transmission and zero transmission exist alternatively. These transmission features result in the periodical reversals of the valley polarization.

It is can be observed that the transmission probability shown in Fig. 6(a) can be explained by the dispersion relation for the superlattice structure shown in Fig. 6(c), and the transmission shown in Fig. 6(b) can be understood from Fig. 6(d). So, the characteristics of the transport properties are determined by the superlattice structure. Since it is easy for us to change the external gate voltage of the superlattice structure, it is very convenient to control the tunneling conductance as well as the valley polarization.

**Figure 7:**
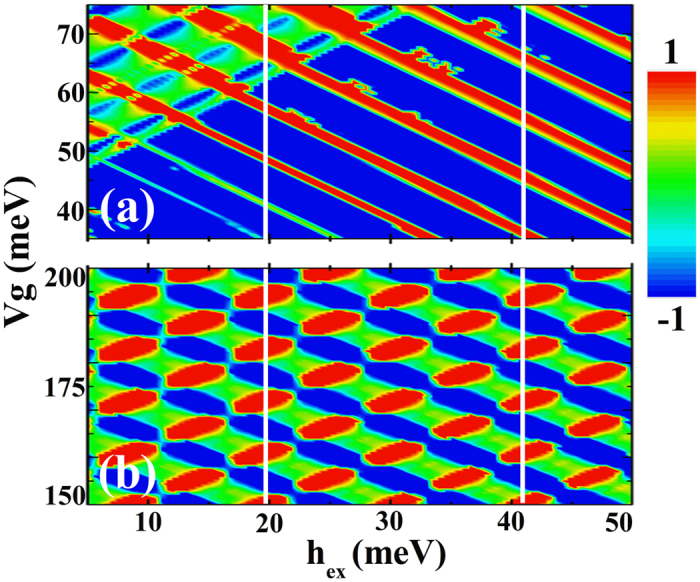
(**a**) Contour plots of the valley polarization as a function of exchange splitting *h*_*ex*_ and external gate voltage. (**b**) Contour plots of the spin polarization as a function of exchange splitting h_ex_ and external gate voltage. Other parameters are the same as that of Fig. 3.

Up to now, the exchange splitting is chosen to be 36 meV in our discussion, and we have not considered any other values. As the value of the exchange splitting is not certain, we need to examine the effects of the exchange splitting on the main results of our study. In Fig. 7, we show the contour plots of valley polarization and spin polarization as a function of the external gate voltage and exchange splitting. We can see that the valley polarization can be switched when the exchange splitting is small, and the switching of the valley polarization does not depend on the value of exchange splitting strongly. The valley polarization can be switched in the whole range of exchange splitting considered: 5~50 meV. However, if the exchange is small, for example, *h*_*ex*_ = 10 *meV*, the switching effect would be better if the external gate voltage is larger: 50~70 meV. In Fig. 7(b), we show the contour plots of the spin polarization as a function of the exchange splitting *h*_*ex*_ and external gate voltage, which shows that the value of the exchange splitting does not affect our main conclusion: spin polarization reversals can be found no matter the exchange splitting is large or small within the considered range. Although we use the value of 36 meV in our numerical calculation, our qualitative prediction is appropriate for a wide region of exchange splitting values.

## Conclusions

In summary, we have investigated the generation of spin and valley polarized current in silicene with a superlattice of ferromagnetic stripes. We calculated the spin and valley resolved conductance of our proposed device, and we found that full spin and valley polarized current can be obtained. The spin and valley polarizations are oscillatory functions of the external gate voltages, and we can easily switch the spin and valley polarization from −100% to 100% by a slight change in the gate voltage. The band structure of our proposed superlattice structure is also studied, which helps to establish a clear understanding of the effects of the superlattice structure on electron transmission. In comparison with the single magnetic barrier studied previously, our proposed finite superlattice structure has the advantage that both the valley and spin polarizations can be easily switched with a very small changes in the gate voltage. This characteristic could be useful in the study of valleytronics and spintronics in silicene nanostructures.

## Additional Information

**How to cite this article**: Zhang, Q. *et al*. Electrically controllable sudden reversals in spin and valley polarization in silicene. *Sci. Rep.* **6**, 33701; doi: 10.1038/srep33701 (2016).

## Figures and Tables

**Figure 1:**
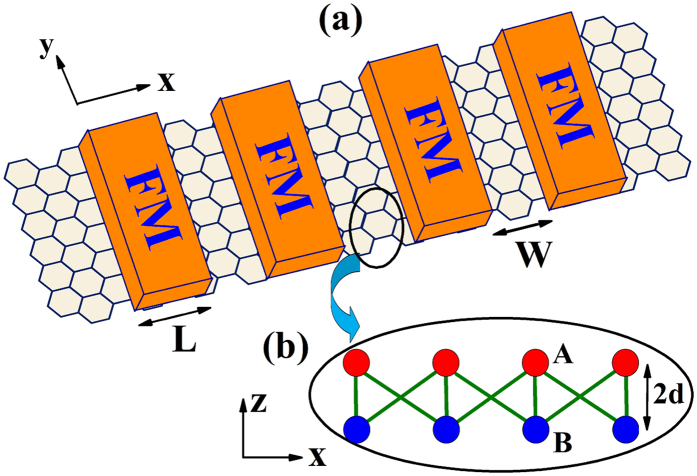
(**a**) Schematic representation of a ferromagnetic superlattice on silicene. (**b**) Side view of silicene vertical buckling. A and B sites of the two sublattices are separated by a perpendicular distance 2d. The ferromagnetic stripes are also used as top gates for adding voltage potentials.
